# Temporal fluxomics reveals oscillations in TCA cycle flux throughout the mammalian cell cycle

**DOI:** 10.15252/msb.20177763

**Published:** 2017-11-09

**Authors:** Eunyong Ahn, Praveen Kumar, Dzmitry Mukha, Amit Tzur, Tomer Shlomi

**Affiliations:** ^1^ Department of Computer Science Technion Haifa Israel; ^2^ Department of Biology Technion Haifa Israel; ^3^ Faculty of Life Sciences Bar‐Ilan University Ramat Gan Israel; ^4^ The Institute of Nanotechnology and Advanced Materials Bar‐Ilan University Ramat Gan Israel; ^5^ Lokey Center for Life Science and Engineering Technion Haifa Israel

**Keywords:** cell cycle, cellular metabolism, isotope tracing, LC‐MS, metabolic flux analysis, Cell Cycle, Genome-Scale & Integrative Biology, Metabolism

## Abstract

Cellular metabolic demands change throughout the cell cycle. Nevertheless, a characterization of how metabolic fluxes adapt to the changing demands throughout the cell cycle is lacking. Here, we developed a temporal‐fluxomics approach to derive a comprehensive and quantitative view of alterations in metabolic fluxes throughout the mammalian cell cycle. This is achieved by combining pulse‐chase LC‐MS‐based isotope tracing in synchronized cell populations with computational deconvolution and metabolic flux modeling. We find that TCA cycle fluxes are rewired as cells progress through the cell cycle with complementary oscillations of glucose versus glutamine‐derived fluxes: Oxidation of glucose‐derived flux peaks in late G1 phase, while oxidative and reductive glutamine metabolism dominates S phase. These complementary flux oscillations maintain a constant production rate of reducing equivalents and oxidative phosphorylation flux throughout the cell cycle. The shift from glucose to glutamine oxidation in S phase plays an important role in cell cycle progression and cell proliferation.

## Supporting information



Appendix

Dataset EV1

Dataset EV2

Dataset EV3

Review Process File
