# Supplementary material for: Temporal fluxomics reveals oscillations in TCA cycle flux throughout the mammalian cell cycle
Source: Mol Syst Biol. 2017 Nov 9;13(11):953. doi: 10.15252/msb.20177763 (PMC5731346; doi:10.15252/msb.20177763)
Supplement: Supplementary file 1 — Appendix [file MSB-13-953-s001.pdf]

Appendix

Table of contents

Figure S1 2
Figure S2 3
Figure S3 4
Figure S4 5
Figure S5 6
Figure S6 7
Figure S7 8
Figure S8 9
Figure S9 10
Figure S10 11
Figure S11 12
Figure S12 13
Figure S13 14
Figure S14 15
Figure S15 16
Figure S16 17
Figure S17 18
Figure S18 19
Figure S19 20
Figure S20 21

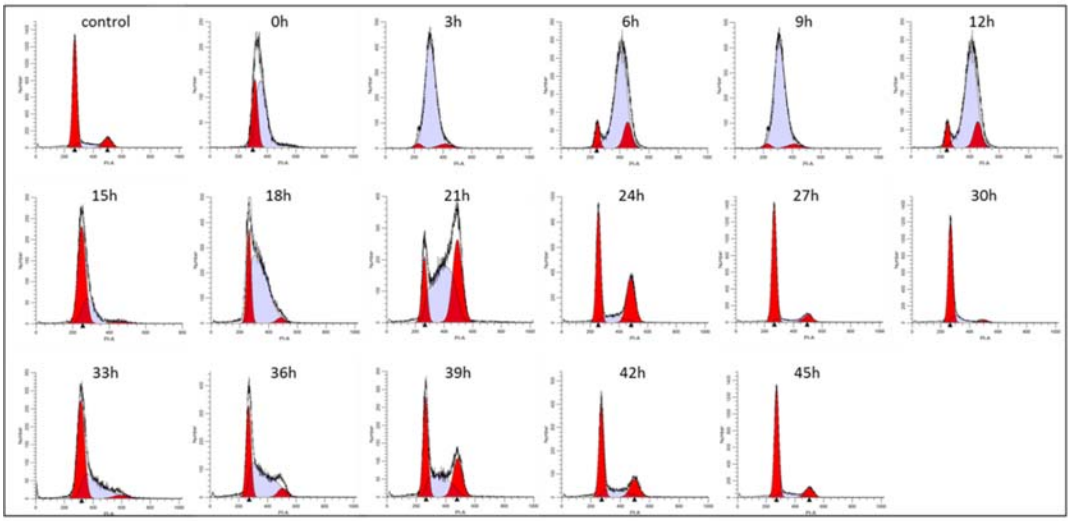

Figure S1: Measurement of cell cycle phase distribution in non-synchronized (control) and synchronized HeLa cells at different times after released from growth arrest, performed by propidium iodide (PI) staining/FACS analysis.

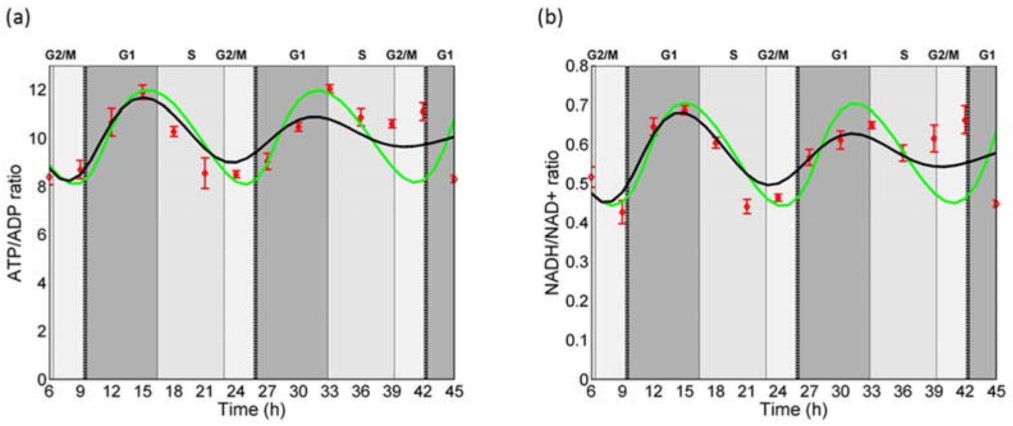

Figure S2: The measured and deconvoluted ATP/ADP ratio (a) and NADH/NAD+ ratio (b). Measured ratios in red (mean and s.d. of n=5); the deconvoluted signal (in case of no synchronization loss) in green; and the expected ratios considering the loss in synchronization in black.

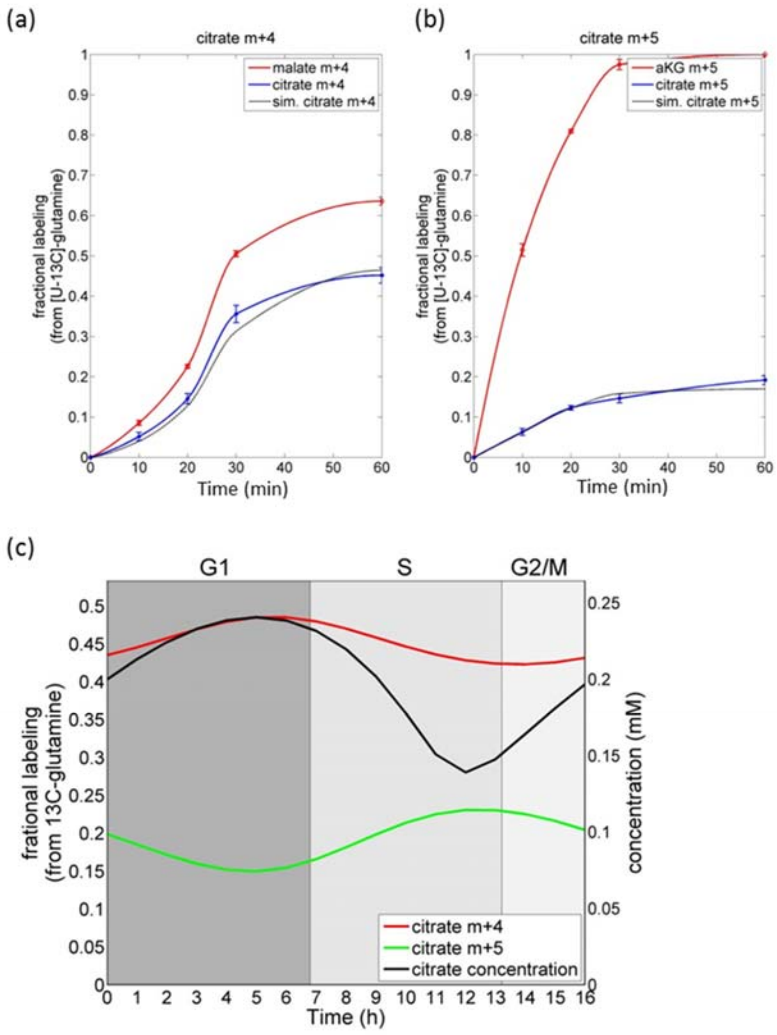

Figure S3: Computational modelling of citrate producing fluxes: Citrate synthase (*v1*) and reductive IDH (*v2*) fluxes. The measured (blue; mean and s.d. of n=3) and simulated (black curve) 1-hour labeling kinetics of citrate m+4 and m+5, as well as the measured labeling kinetics of malate m+4 (red; producing citrate m+4 via *v1*) and α-ketoglutarate m+5 (producing citrate m+5 via *v2*) in non-synchronized cells fed with [U-^{13}C]-glutamine (a-b). Estimating the flux through *v1* and *v2*, in non-synchronized cells, the simulated labeling kinetics of citrate m+4 and m+5 match the measured labeling kinetics. (c) The deconvoluted fractional labeling of citrate m+4 and m+5 after 1-hour feeding with [U-^{13}C]-glutamine and citrate concentration throughout the cell cycle.

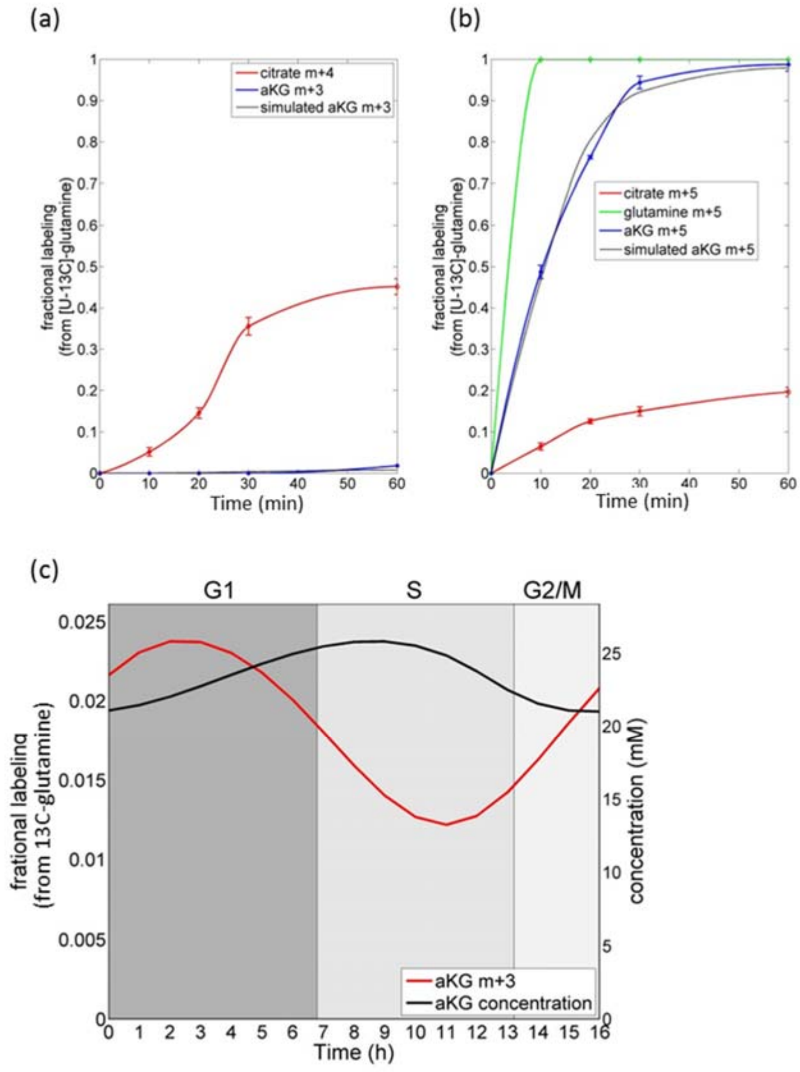

Figure S4: Computational modelling of fluxes producing α-ketoglutarate/glutamate: Glutamine->glutamate (v8) and oxidative IDH (v3) fluxes. The measured (blue; mean and s.d. of n=3) and simulated (black curve) 1-hour labeling kinetics of α-ketoglutarate m+3 and m+5, as well as the measured labeling kinetics of citrate m+4 (producing α-ketoglutarate m+3 via *v3*), citrate m+5 (producing α-ketoglutarate m+5 via *v3*), and glutamine (producing α-ketoglutarate m+5 via *v8*) in non-synchronized cells fed with [U-^{13}C]-glutamine (a-b). Estimating the flux through *v3* and *v8*, in non-synchronized cells, the simulated labeling kinetics of α-ketoglutarate m+3 and m+5 match the measured labeling kinetics. (c) The deconvoluted fractional labeling of α-ketoglutarate m+3 after 1-hour feeding with [U-^{13}C]-glutamine and α-ketoglutarate concentration throughout the cell cycle.

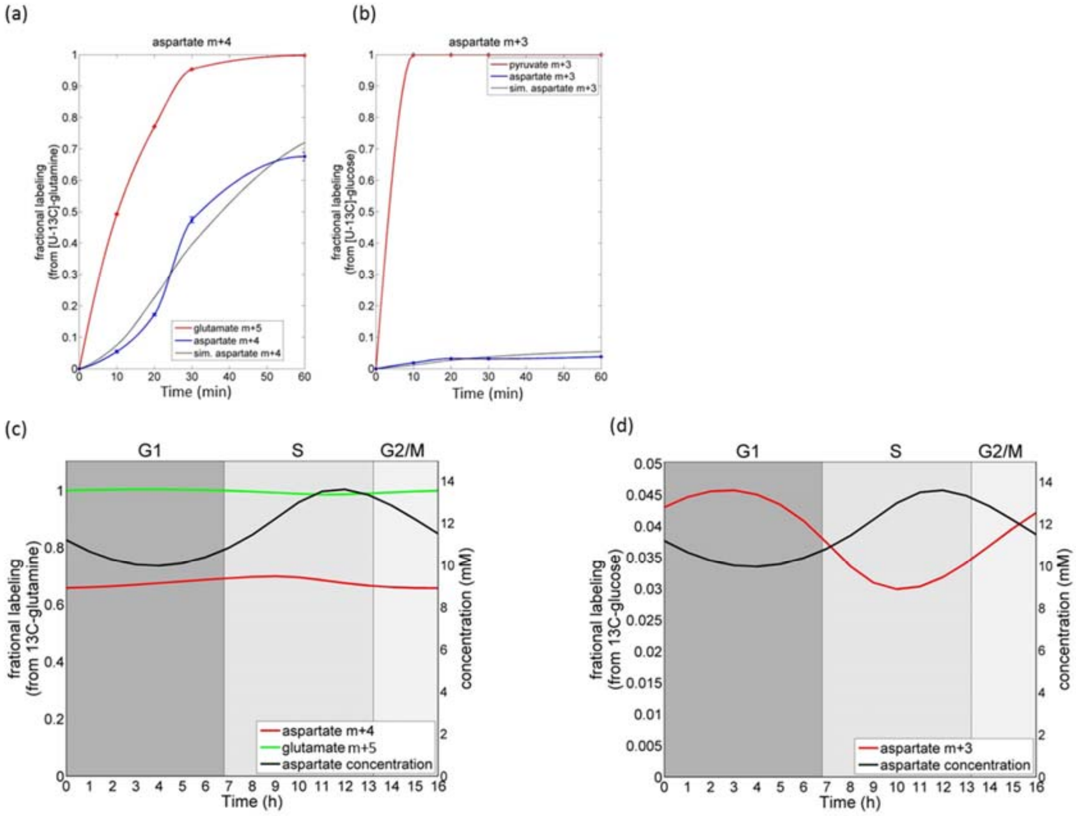

Figure S5: Computational modelling of malate/aspartate producing fluxes: α-ketoglutarate oxidation (*v4*) and pyruvate carboxylase (*v7*) fluxes. The measured (blue; mean and s.d. of n=3) and simulated (black curve) 1-hour labeling kinetics of aspartate m+4, and the isotopic labelling kinetics of glutamate m+5 (producing aspartate m+4 via *v4*), in non-synchronized cells fed with [U-^{13}C]-glutamine. (b) The measured (blue; mean and s.d. of n=3) and simulated (black curve) 1-hour labeling kinetics of aspartate m+3, and the isotopic labelling kinetics of pyruvate m+3 (producing aspartate m+3 via *v7*), in non-synchronized cells fed with [U-^{13}C]-glucose. (c) The deconvoluted fractional labeling of aspartate m+4 and glutamate m+5 after 1-hour feeding with [U-^{13}C]-glutamine, and aspartate concentration throughout the cell cycle. (d) The deconvoluted fractional labeling of aspartate m+3 after 1-hour feeding with [U-^{13}C]-glucose and aspartate concentration throughout the cell cycle.

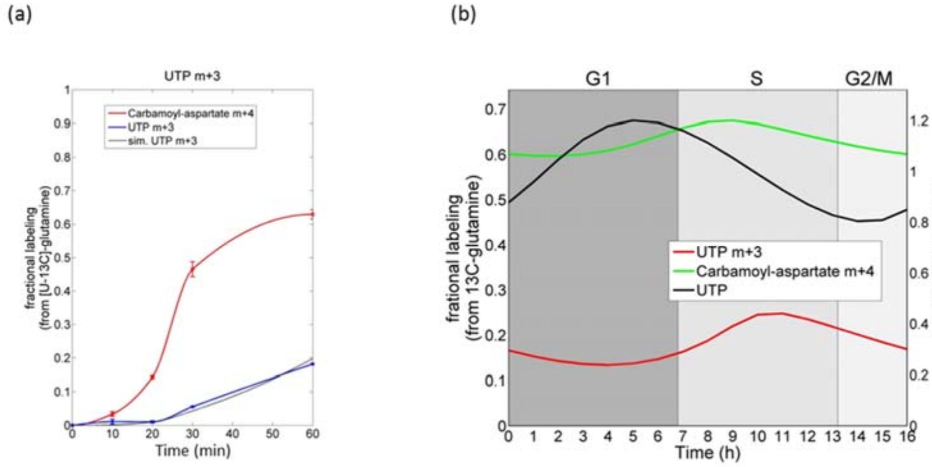

Figure S6: Computational modelling of the UTP producing flux (*v5*). (a) The measured (blue; mean and s.d. of n=3) and simulated (black curve) 1-hour labeling kinetics of UTP m+3 and measured labeling kinetics of carbamoyl-aspartate m+4 (producing UTP m+3 via *v5*), in non-synchronized cells fed with [U-^{13}C]-glutamine. Estimating the flux through v5 in non-synchronized cells, the simulated labeling kinetics of UTP m+3 matches the measured labeling kinetics. (b) The deconvoluted fractional labeling of UTP m+3 and carbamoyl-aspartate m+4 after 1-hour feeding with [U-^{13}C]-glutamine and UTP concentration throughout the cell cycle.

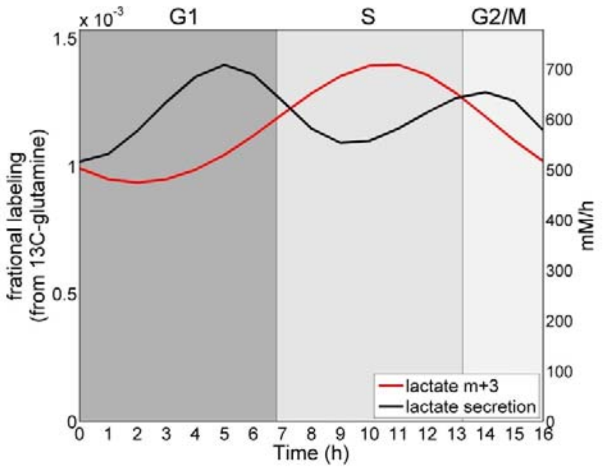

Figure S7: Computational modelling of malic enzyme flux (*v6*). The deconvoluted fractional labeling of lactate m+3 after 1-hour feeding with [U-^{13}C]-glutamine throughout the cell cycle (red); and lactate secretion flux throughout cell cycle (black).

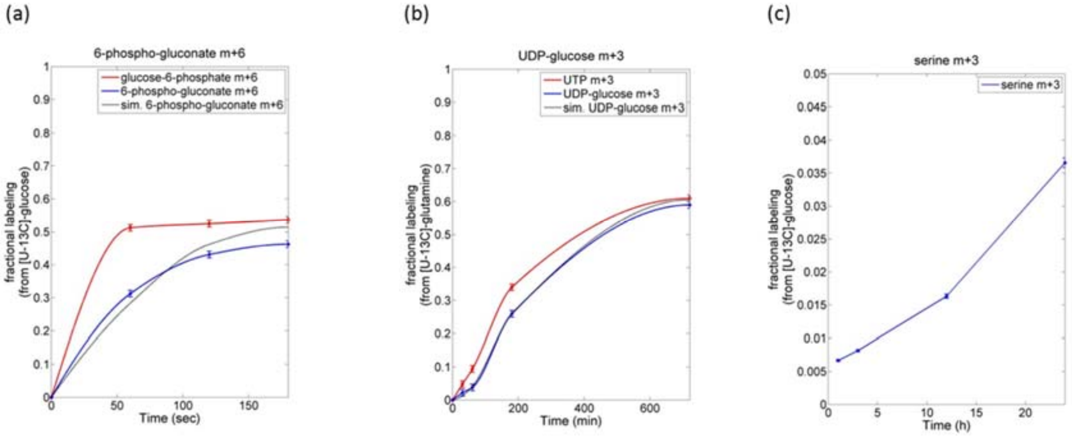

Figure S8: Quantifying metabolic flux through pathways branching out of glycolysis in non-synchronized cells. (a) Oxidative PPP flux is estimated based on the rate of labeling of 6-phospho-gluconate from glucose-6-phosphate, when feeding isotopic glucose (considering a 6-phospho-gluconate concentration of 0.04mM). Reductive PPP flux is roughly similar to oxidative PPP considering that the steady state m+1 labeling of ribose converges to ~50% when feeding [1,2-^{13}C]-glucose (not shown). (b) Glycogenesis flux is estimated based on the rate of labeling of UDP-glucose m+3 from UTP m+3, when feeding isotopic glutamine (considering a UDP-glucose concentration of 0.26mM). (c) The fractional labeling of serine m+3 is less than 5% after feeding isotopic glucose for 24h; hence *de novo* serine biosynthesis is less than 5% of the total serine consumption rate from media (<10mM/h).

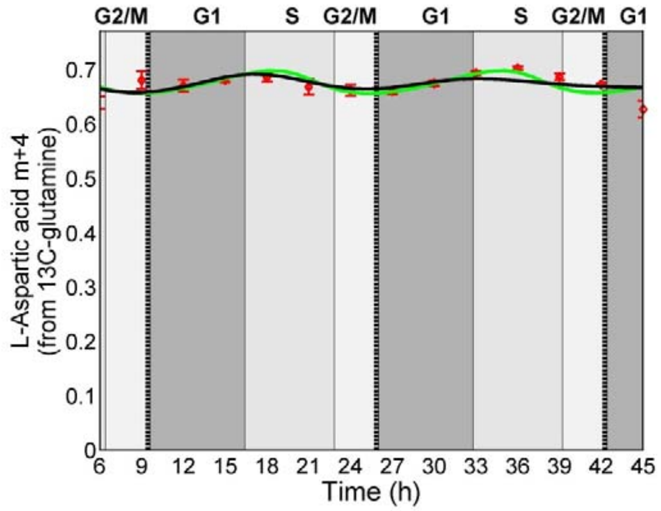

Figure S9: The cell cycle kinetics of L-aspartate m+4 when feeding synchronized HeLa cells with [U-^{13}C]-glutamine for one hour. Measured ratios in red (mean and s.d. of n=3); the deconvoluted signal (in case of no synchronization loss) in green; and the expected ratios considering the loss in synchronization in black.

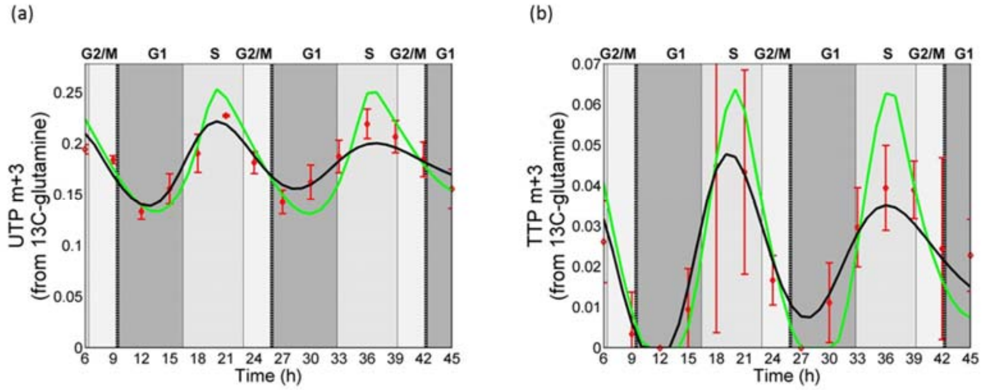

Figure S10: The cell cycle kinetics of UTP m+3 and TTP m+3 when feeding synchronized HeLa cells with [U-^{13}C]-glutamine for one hour. Measured ratios in red (mean and s.d. of n=3); the deconvoluted signal (in case of no synchronization loss) in green; and the expected ratios considering the loss in synchronization in black.

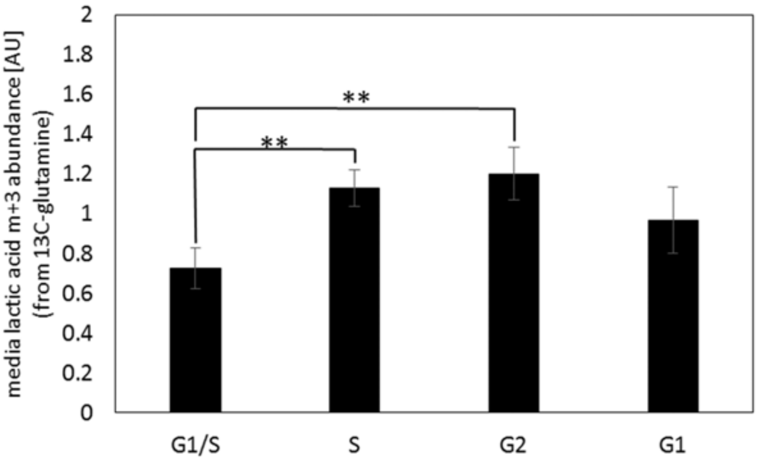

Figure S11: The measured abundance of lactate m+3 in media samples when feeding synchronized cells with [U-^{13}C]-glutamine for three hours. ** indicates *p-value* from Student's t-test ≤ 0.01.

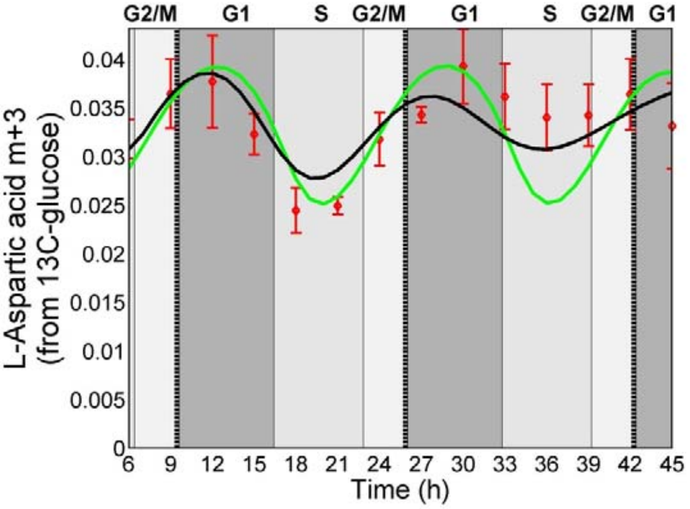

Figure S12: The cell cycle kinetics of L-aspartate m+3 when feeding synchronized HeLa cells with [U-^{13}C]-glucose for one hour. Measured ratios in red (mean and s.d. of n=3); the deconvoluted signal (in case of no synchronization loss) in green; and the expected ratios considering the loss in synchronization in black.

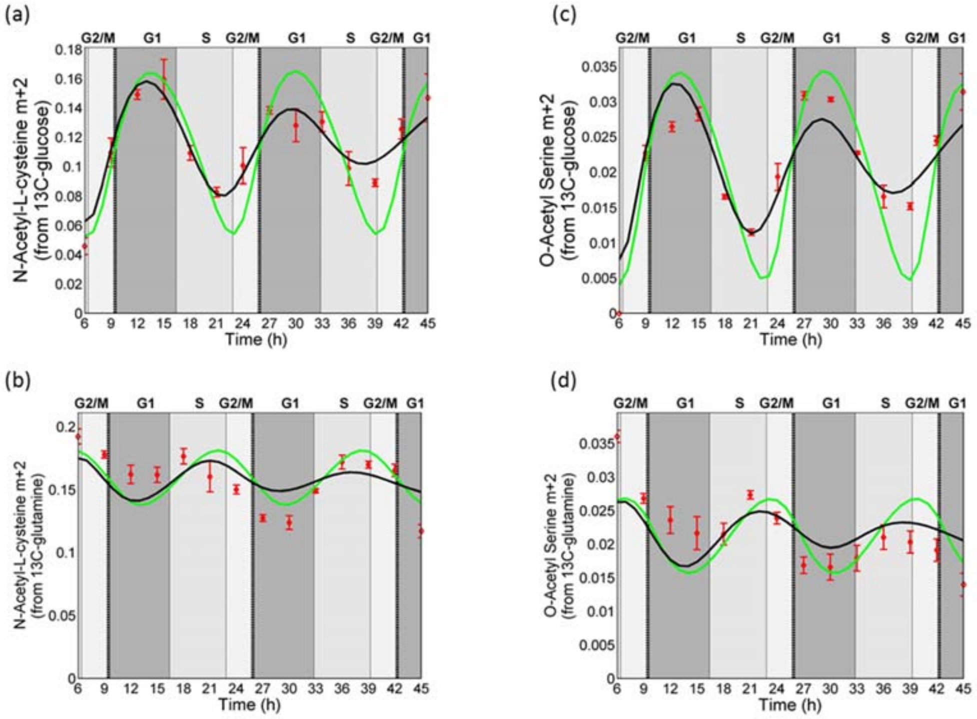

Figure S13: Oscillations in m+2 labeling of N-acetyl-cysteine (a-b) and O-acetyl-serine (c-d) when feeding isotopic glucose (a and c) and isotopic glutamine (b and d). Measured ratios in red (mean and s.d. of n=3); the deconvoluted signal (in case of no synchronization loss) in green; and the expected ratios considering the loss in synchronization in black.

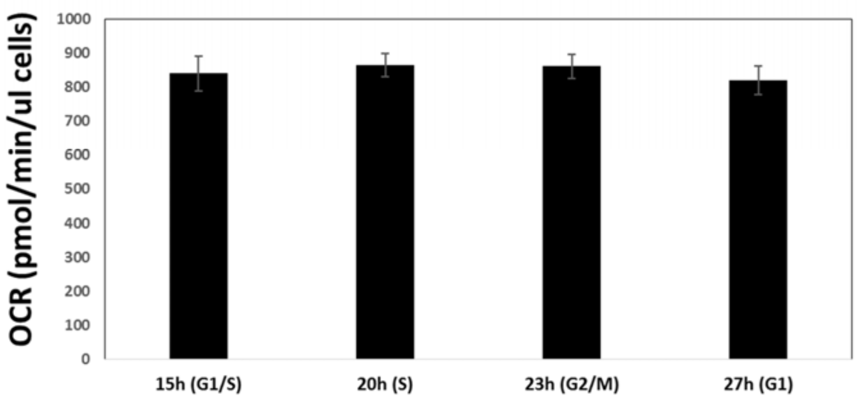

Figure S14: Oxygen consumption used for oxidative phosphorylation in synchronized HeLa cells, measured using a Seahorse XFp Flux Analyzer (the non-mitochondrial oxygen consumption after treatment with the ETC inhibitors rotenone and antimycin A subtracted from the basal OCR). No significant differences were found in the rate of oxygen consumption in different cell cycle phase. Data have been represented in averages ± SD from n=3 replicates).

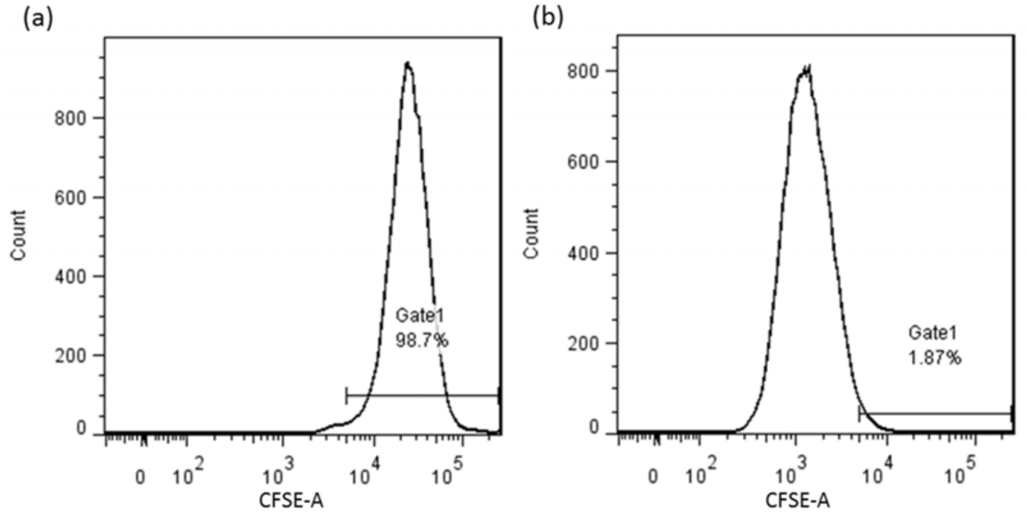

Figure S15: FACS measurement of CFSE signal in non-synchronized HeLa cells immediately after feeding to cells (a) and after 72 hours (b).

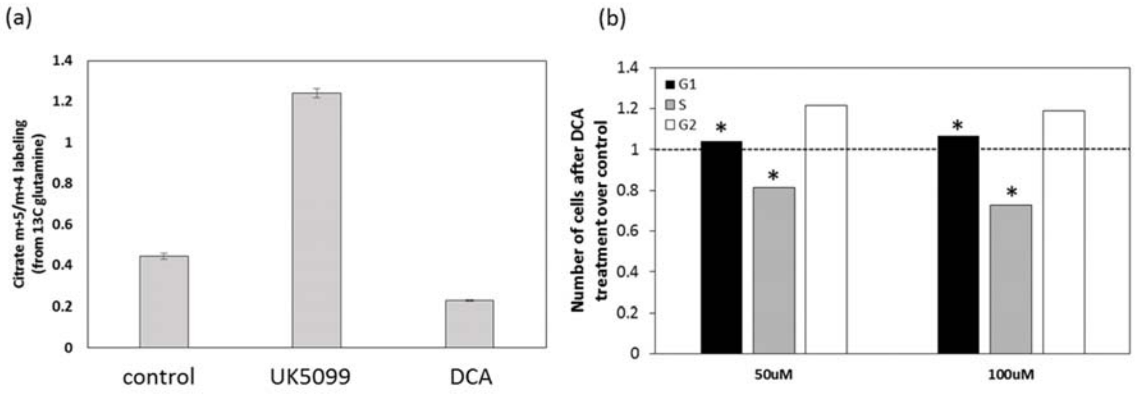

Figure S16: (a) Citrate m+5/m+4 ratio when feeding non-synchronized HeLa cells with an inhibitor of the mitochondrial pyruvate transporter (UK5099) and when feeding DCA. (b) The fraction of cells in G1, S, and G2/M phases in non-synchronized HeLa cells after 24 hour treatment with UK5099 (normalized by measurements in untreated control cells).

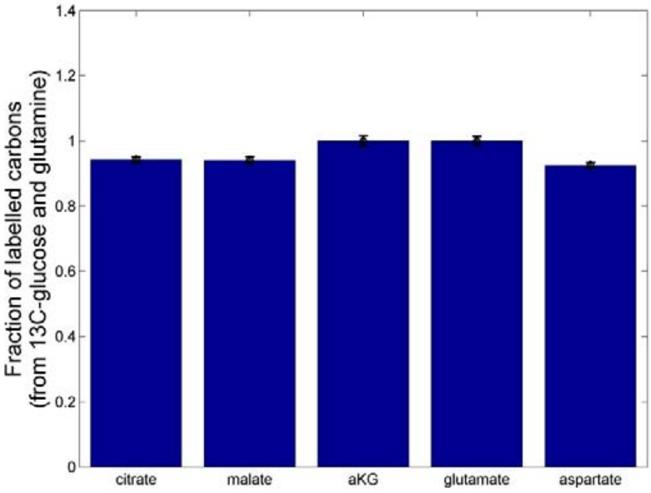

Figure S17: Fraction of carbons labeled in TCA cycle intermediates and other metabolites when feeding both [U-^{13}C]-glucose and [U-^{13}C]-glutamine for 24h. Additional carbons are derived from the fixation of atmospheric CO_2 through reductive isocitrate dehydrogenase (IDH) and pyruvate carboxylase (PC). E.g. for citrate, the fraction of citrate m+5 when feeding isotopic glutamine (as an indication of reductive IDH activity) and malate m+3 when feeding isotopic glucose (as an indication pyruvate carboxylase activity) suggest that another ~3% of the citrate carbons are derived from CO_2 fixation.

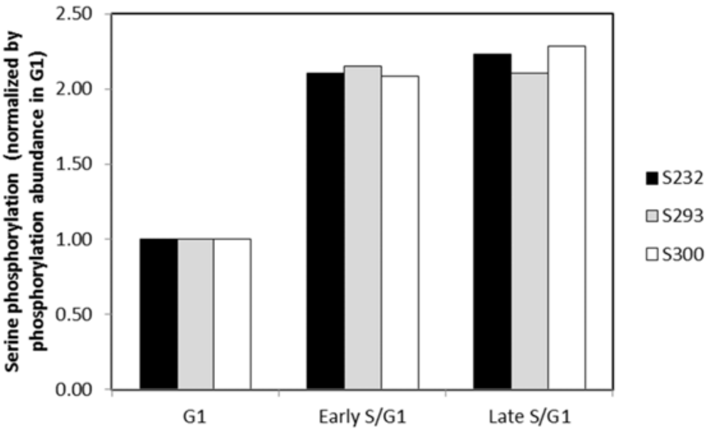

Figure S18: Abundance of PDH E1 phosphorylation increases in early and late S versus in G1 in all three reported phosphorylation sites (data from Olsen, J. V, *Sci Signal* **3,** 2010).

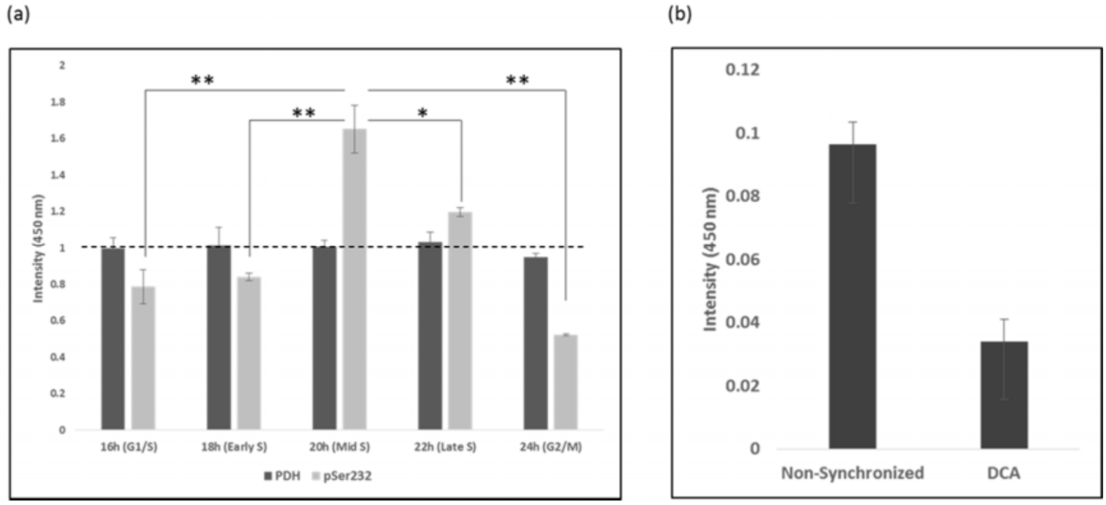

Figure S19: (a) Phosphorylation of PDH Ser232 increases in S phase. HeLa cells were synchronized using double thymidine block and lysates were prepared at different stages of the cell cycle. PDH phosphorylation was measured via an ELISA kit. All experiments were done in triplicates; two tailed Student's T-test was applied to calculate *p*-values. Notably, the concentration of PDH (measured via an ELISA kit) remains constant throughout the cell cycle. (b) DCA treatment significantly decreases PDH phosphorylation at Ser232.

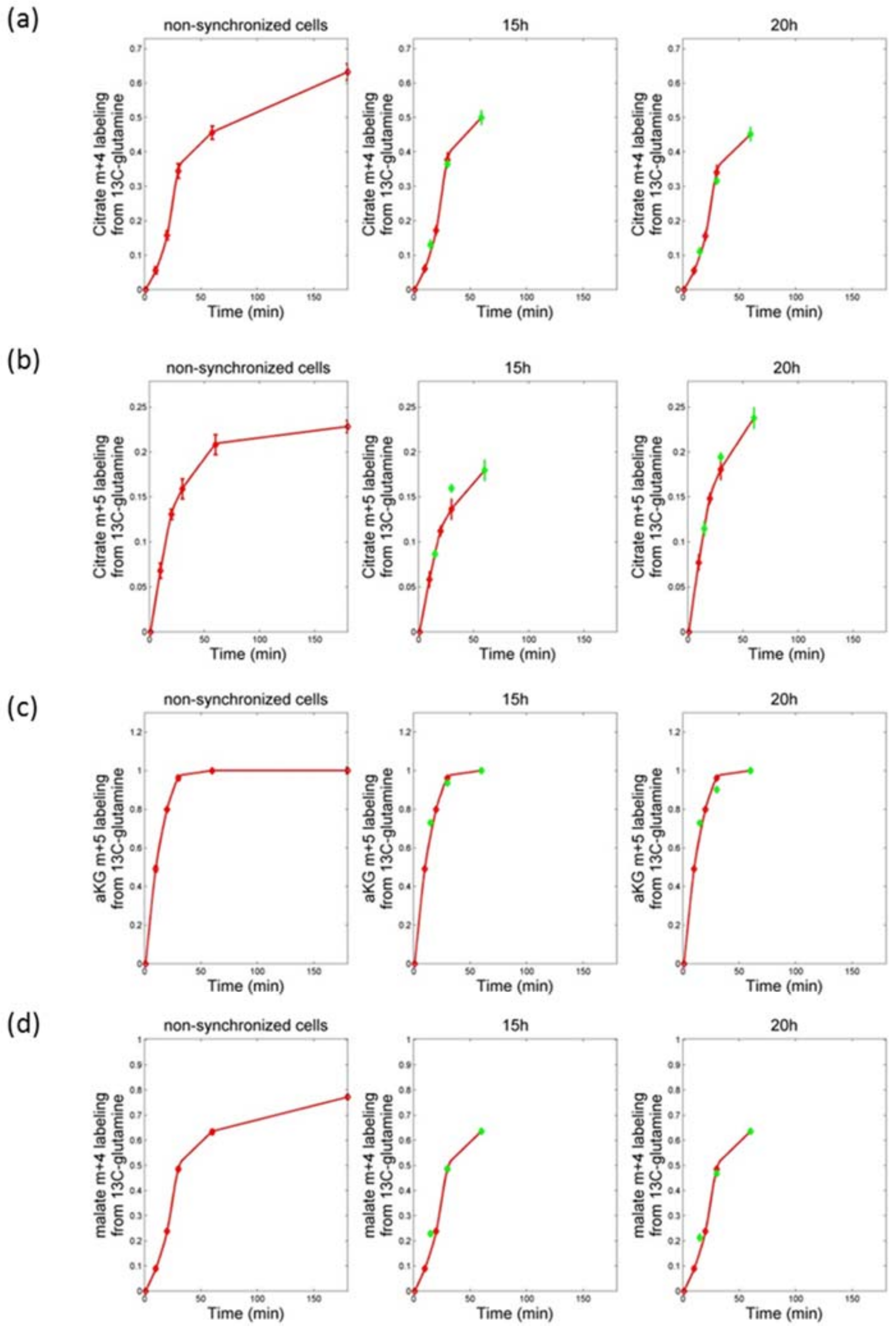

Figure S20: (a) Measured (green dots) versus estimated (red curves) labeling kinetics of citrate m+4 (a), citrate m+5 (b), α-ketoglutarate m+5 (c), and malate m+4 (d), when feeding non-synchronized and synchronized cells in G1/S (15h) and S (20h) with isotopic glutamine. The estimated labeling kinetics in the synchronized cells was derived based on Eq. 13 (in main text).
